# Effect of Grafting on Viral Resistance of Non-transgenic Plum Scion Combined With Transgenic PPV-Resistant Rootstock

**DOI:** 10.3389/fpls.2021.621954

**Published:** 2021-02-01

**Authors:** Tatiana Sidorova, Dmitry Miroshnichenko, Ilya Kirov, Alexander Pushin, Sergey Dolgov

**Affiliations:** ^1^Branch of Shemyakin and Ovchinnikov Institute of Bioorganic Chemistry, Russian Academy of Science, Pushchino, Russia; ^2^All-Russia Research Institute of Agricultural Biotechnology, Russian Academy of Science, Moscow, Russia; ^3^Federal Horticulture Center for Breeding, Agrotechnology and Nursery, Moscow, Russia

**Keywords:** stone fruits, genetic transformation, transgrafting, RNA interference, hpRNA, mobile sRNA, *Plum pox virus*

## Abstract

In stone fruit trees, resistance to *Plum pox virus* (PPV) can be achieved through the specific degradation of viral RNA by the mechanism of RNA interference (RNAi). Transgenic virus-resistant plants, however, raise serious biosafety concerns due to the insertion and expression of hairpin constructs that usually contain various selective foreign genes. Since a mature stone tree represents a combination of scion and rootstock, grafting commercial varieties onto transgenic virus-tolerant rootstocks is a possible approach to mitigate biosafety problems. The present study was aimed at answering the following question: To what extent are molecular RNAi silencing signals transmitted across graft junctions in transgrafted plum trees and how much does it affect PPV resistance in genetically modified (GM)/non-transgenic (NT) counterparts? Two combinations, NT:GM and GM:NT (scion:rootstock), were studied, with an emphasis on the first transgrafting scenario. Viral inoculation was carried out on either the scion or the rootstock. The interspecific rootstock “Elita” [(*Prunus pumila* L. × *P. salicina* Lindl.) × (*P. cerasifera* Ehrh.)] was combined with cv. “Startovaya” (*Prunus domestica* L.) as a scion. Transgenic plum lines of both cultivars were transformed with a PPV-coat protein (CP)-derived intron-separate hairpin-RNA construct and displayed substantial viral resistance. High-throughput sequence data of small RNA (sRNA) pools indicated that the accumulation of construct-specific small interfering RNA (siRNA) in transgenic plum rootstock reached over 2%. The elevated siRNA level enabled the resistance to PPV and blocked the movement of the virus through the GM tissues into the NT partner when the transgenic tissues were inoculated. At the same time, the mobile siRNA signal was not moved from the GM rootstock to the target NT tissue to a level sufficient to trigger silencing of PPV transcripts and provide reliable viral resistance. The lack of mobility of transgene-derived siRNA molecules was accompanied by the transfer of various endogenous rootstock-specific sRNAs into the NT scion, indicating the exceptional transitivity failure of the studied RNAi signal. The results presented here indicate that transgrafting in woody fruit trees remains an unpredictable practice and needs further in-depth examination to deliver molecular silencing signals.

## Introduction

Nowadays, commercial stone fruit trees are almost exclusively grafted. Primarily implemented as a method of accelerated propagation, grafting now achieves various goals in modern horticulture (Warschefsky et al., 2016). The proper combination of scion and rootstock, sometimes belonging to various species, enhances the productivity of fruit trees and increases the tolerance/resistance to biotic and abiotic stresses. Grafting also improves fruit quality and provides new superior or dwarf architecture to meet a variety of practical needs (Rugini et al., 2016). The interaction between scion and rootstock is bi-directional. Various constituents, including ions, nutrients, hormones, peptides/proteins, small organic molecules, and nucleic acids, have been shown to move across the graft union through the phloem and xylem (Albacete et al., 2015; Lu et al., 2020). As a result, grafting triggers new systemic signals that are able to interact with genes involved in metabolic processes, hormone signaling, the activity of transcription factors, physiological responses to environmental stimuli, and others (Albacete et al., 2015; Warschefsky et al., 2016; Lu et al., 2020).

Managing the rootstock-to-scion mobility of specific functional compounds is of particular interest, especially in the context of the rapid development of transgenic technologies. The entrance of genetically modified (GM) crops on the global agricultural market led to the emergence of a new scion:rootstock scenario named transgrafting. Transgrafting is defined as the combination of non-transgenic (NT) scion with genetically engineered (transgenic) rootstock, or vice versa (Song et al., 2015). Certainly for woody fruit species, the main transgrafting strategy is to use GM rootstock to support NT scion. Such transgrafted trees are primarily intended to increase the food security of biotech crops since the fruits harvested from a non-transgenic scion remain genetically unmodified (Lemgo et al., 2013). At the same time, fruit growers can benefit directly from the improved properties of GM rootstock, or indirectly from the transgene-derived molecular signals transmitted into the scion through grafting (Song et al., 2015). Additionally, the use of transgrafted plants can minimize concerns of transgene flow, as no transgenic pollen will arise from the floriferous NT scion. The reverse transgrafting strategy (GM scion on NT rootstock) is not as much of an interest for fruit growing, but it offers potential benefits from the cultivation of herbaceous root or tuber crops (Kyriacou et al., 2017).

The transgrafting scenario has already been applied to various perennial fruit crops, including apple (Smolka et al., 2010; Flachowsky et al., 2012; Artlip et al., 2016), sweet cherry (Zhao and Song, 2014; Rugini et al., 2015), grape (Vigne et al., 2004; Dandekar et al., 2019), plum (Nagel et al., 2010), walnut (Vahdati et al., 2002; Haroldsena et al., 2012; Liu et al., 2017), sweet orange (La Malfa et al., 2011), and blueberry (Song et al., 2019). The movement of transgenic components, such as RNAs, proteins, and phytohormones, from rootstock to scion has provided disease resistance in grape (Agüero et al., 2005; Dandekar et al., 2019), increased tolerance to viral infection in grape and sweet cherry (Vigne et al., 2004; Zhao and Song, 2014), altered plant size and morphology in sweet orange and apple (Smolka et al., 2010; La Malfa et al., 2011), and stimulated early flowering in blueberry (Song et al., 2019). On the other hand, mechanisms regulating the transmission of various transgene-derived signals are not completely understood. Various attempts to achieve transgraft-mediated effects in recipient scions were unsuccessful since the long-distance transmission of transgene-derived signals was not always detected in transgrafted woody plants. No translocation of *RolB* transcripts from transgenic rootstock to wild-type (WT) scion was found in apple (Smolka et al., 2010). Transgenic plum rootstock expressing the gene encoding Gastrodia antifungal protein (GAFP-1) was unable to translocate transgenic protein into NT scion (Nagel et al., 2010). No graft transmission of transgene-derived double-stranded RNA (dsRNA) was observed in mature apple plants (Flachowsky et al., 2012). In walnut, the trafficking of transgene-derived DNA, protein, and mRNA across the GM graft was not observed in vegetative tissues (Haroldsena et al., 2012). Besides, the transgrafting did not provide additional benefits, since a small amount of transferred content was found in the WT scions, sometimes only in specific tissues (Flachowsky et al., 2012; Haroldsena et al., 2012; Liu et al., 2017). Discrepancies in the mobility of transgenic components observed from one study to another indicate the complexity of transgene-derived signal trafficking. For this reason, it is not yet possible to accurately predict the real effect of planned transgrafting and it should be explored experimentally, considering the specifics of plant species, genetic construct, molecular targets, physiological behavior, etc.

In the present study, we developed transgrafted plum trees with the aim to increase the resistance of non-transgenic tissue to Sharka disease through signaling mediated by transgene-derived small interfering RNA (siRNA). Plum, like many other stone fruit trees, seriously suffers from attacks of *Plum pox virus* (PPV), the causal agent of Sharka disease (Scorza et al., 2013). PPV is a single-stranded RNA virus belonging to the *Potyviridae* family that is currently recognized as a quarantine pathogen easily transmitted by aphids in a non-persistent manner and by grafting. PPV has spread to all “green” continents and causes enormous losses in commercial orchards and private gardens. It affects the fruits of commercially cultivated stone fruits such as plums (*P. domestica*, *P. salicina*), apricots (*P. armeniaca*), cherries (*P. avium* and *P. cerasus*), peaches and nectarines (*P. persica*), almonds (*P. dulcis*), and other ornamental and wild *Prunus* species (Scorza et al., 2013). Unfortunately, *Prunus* species lack the natural genes for resistance, so interspecific grafting also fails to protect commercial orchards from PPV infection.

At the same time, the combination of modern genetic transformation methods with RNA interference (RNAi) technology may provide reliable viral resistance in plant species, while the original characteristics of transformed cultivars remain unchanged. Effective virus suppression could be achieved at the transcriptional level through the production of dsRNA by RNAi-eliciting constructs (Khalid et al., 2017; Limera et al., 2017). Currently, the most successful approach relies on the use of inverse repeat sequences from the viral genome to produce an abundant amount of hairpin dsRNA interfering with viral replication (Khalid et al., 2017). Expression of various transgenic construct-encoding PPV-derive self-complementary hairpin RNA (hpRNA) and intron-spliced hpRNA has been shown to successfully provide long-time resistance to various PPV strains in transgenic plum (Ilardi and Tavazza, 2015; Petri et al., 2018). An important characteristic of RNAi is the short- and long-distance mobility of the silencing signal through the graft junction (Kehr and Kragler, 2018). This rootstock-to-scion transitivity was found to be associated with mobile siRNA, which is formed in cells through cleavage of dsRNA (Liu and Chen, 2018; Choudhary et al., 2019; Zhang et al., 2019). Numerous studies revealed extensive exchange of endogenous and exogenous small RNA (sRNA), including siRNA and micro RNA (miRNA), in woody and herbaceous plant species between intra- and intergeneric grafts (Pyott and Molnar, 2015; Kyriacou et al., 2017; Kehr and Kragler, 2018; Liu and Chen, 2018; Choudhary et al., 2019; Zhang et al., 2019; Lu et al., 2020). This gives rise to an opportunity to design transgrafted plum trees eliciting an abundant amount of dsRNA/siRNA mobile signals in the transgenic rootstock to achieve PPV resistance in a scion. An important feature of such virus-resistant transgraft plants is that no foreign proteins will be produced and, therefore, delivered to the fruit, since the post-transcriptional silencing of viral genes is triggered by dsRNA (Lemgo et al., 2013).

Several reports have been published over the past decade describing some success in controlling viral resistance in transgrafted herbaceous and woody species. In tobacco (*Nicotiana benthamiana*), grafting onto transgenic stock expressing *Potato spindle tuber viroid*-derived hpRNAs attenuated viroid accumulation in NT scion (Kasai et al., 2013). Similarly, the RNAi silencing signal has been proven to communicate between transgenic tobacco rootstock and NT scion, and to confer viral resistance due to siRNA-mediated reduction of transcripts of endogenous tobacco genes NtTOM1 and NtTOM3 recruited by tobamovirus to support multiplication (Md Ali et al., 2013). The transgrafting of sweet cherry “Gisela 6” (*P. cerasus* × *P. canescens*), a species closely related to plum from the *Prunus* genus, on transgenic rootstock expressing hairpin sRNA specific to the genomic sequence of *Prunus necrotic ringspot virus* (PNRSV) resulted in increased vitality of NT scion (Zhao and Song, 2014). Resistance to PNRSV was associated with an increased amount of 24 nt siRNA, sourced from the GM rootstock producing it in large quantities. In grapevine (*Vitis vinifera*), grafting onto transgenic lines expressing the coat protein gene from *Grapevine fanleaf virus* (GFLV) resulted in partial resistance to a natural viral infection in some non-transgenic scions; however, no correlation between resistance and transgene mRNA accumulation or CP expression level has been found among resistant and susceptible transgenic lines (Vigne et al., 2004).

Thus, to achieve viral resistance through graft delivery of transgene-derived RNAi-mediated signals, we focused on designing transgrafted composite plum trees that mimic conventional fruit trees consisting of plum cultivar and interspecific rootstock. Previously, we generated transgenic plum cv. “Startovaya” (*Prunus domestica* L.) that overexpressed hairpin RNAi construct containing self-complementary intron-spliced fragments of the PPV-CP gene sequence (Dolgov et al., 2010; Sidorova et al., 2019). As evaluated by regular visual inspections and molecular diagnosis, all tested transgenic GM plum trees showed stable resistance over 10 years after artificial inoculation with PPV (Sidorova et al., 2019). Using the same RNAi construct, we recently generated transgenic plants of interspecific plum rootstock “Elita” [(*Prunus pumila* L. × *P. salicina* Lindl.) × (*P. cerasifera* Ehrh.)]. Although the produced transgenic events have been molecularly characterized (Sidorova et al., 2018), no data concerning the resistance of transgenic plum rootstock to PPV were available. To understand whether the RNAi silencing signals can move from transgenic rootstocks to WT scions and contribute to resistance, we challenged transgenic plants of the plum rootstock with PPV and confirmed their ability to withstand the viral attack. After that, we conducted grafting experiments, produced various non-transgenic and transgenic scion:rootstock combinations, infected plants with PPV, analyzed viral resistance, and sequenced and then profiled PPV-specific siRNA in samples of GM and NT tissues. We found that transgrafting was not successful in promoting PPV resistance in non-transgenic scions, as few siRNA reads corresponding to the hpRNA construct were discovered. In contrast, an abundant transgene-derived siRNA population was detected in GM rootstock correlating with robust viral resistance.

## Materials and Methods

### Transgrafting

The plant material for transgrafting consisted of the semi-dwarf rootstock “Elita” [(*Prunus pumila* L. × *P. salicina* Lindl.) × (*P. cerasifera* Ehrh.)] and the cultivar “Startovaya” (*Prunus domestica* L.). All transgenic material of “Startovaya” was derived from the event RNAi1, which was described in our previous study (Sidorova et al., 2019). The transgenic plant material of rootstock “Elita” was derived from events RNAi1 and RNAi2, which were generated in our previous study (Sidorova et al., 2018) and characterized for virus resistance in the present study. All transgenic events successfully expressed the hpRNA construct consisting of the self-complementary fragments (698 bp) of the CP gene sequence of PPV-D (NCBI accession number: D13751.1) separated by a PDK intron, under the modified CaMV 35S promoter (Dolgov et al., 2010).

**Figure 1:**
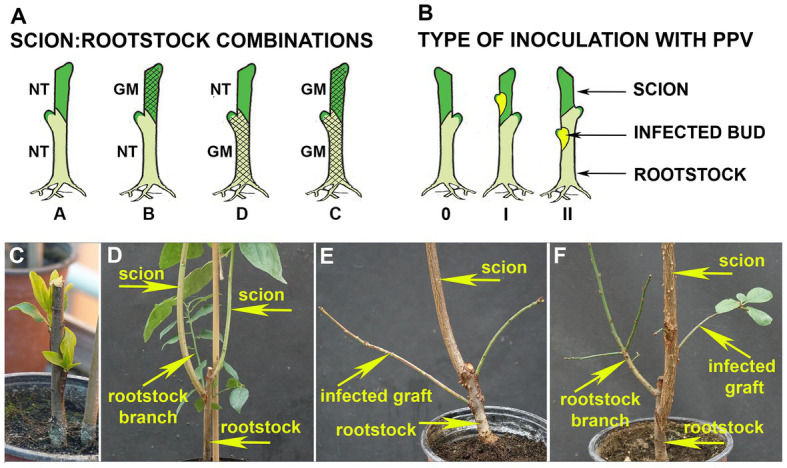
Transgrafting experiments in plum. **(A)** Schematic illustration of four scion:rootstock (“Startovaya”: “Elita”) combinations which involved transgenic (GM) and non-transgenic (NT) plant material. Transgenic lines of “Startovaya” and “Elita” used for transgrafting expressed hairpin RNA (hpRNA) construct consisting of self-complementary intron-separated fragments of the *Plum pox virus* (PPV) coat protein (CP) gene sequence driven by enhanced 35S promoter. **(B)** Types of viral inoculation of composite plum trees with PPV-infected buds. The individual trees of all scion:rootstock combinations were inoculated by T-budding on either scion (I) or rootstock (II); uninfected trees were left for comparative tests (0). **(C)** Transgenic rootstock “Elita” grafted with non-transgenic “Startovaya” buds; 2-year-old plant after chilling period. **(D)** Transgrafted plum at the end of vegetative season consisting of surviving rootstock branch and two well-developed scion branches grown from grafted “Startovaya” buds. **(E)** Transgrafted tree (GM scion:NT rootstock) with two infected shoots (“grafts”) grown out from PPV-positive buds grafted on the rootstock, end of third vegetative season. **(F)** Transgrafted tree consisting of the three types of branches representing scion (NT “Startovaya”), rootstock (GM “Elita”), and infectious grafts grown from PPV-infected bud on scion.

Four scion:rootstock (“Startovaya”: “Elita”) combinations were designed involving transgenic (noted as GM) and non-transgenic (noted as NT) plant material (Figure 1A). Transgraft combinations were included the conjunction of transgenic “Startovaya” as scions with NT “Elita” as rootstocks (GM:NT, combination B); conversely, NT scions were also grafted on transgenic rootstocks (NT:GM, combination D). Additionally, non-transgenic (NT:NT, combination A) and transgenic (GM:GM, combination C) scion:rootstock pairs were produced. All NT and GM rootstock plants were obtained from *in vitro* virus-free shoots. They were cultured, rooted, and transplanted to a greenhouse as described (Sidorova et al., 2017), and grown during 2017. In August 2018, the 1.5-year-old rootstock shoots were grafted by T-budding with the appropriate GM or NT scion using a standard piece of stem with an axillary bud, taken from budwoods of the same age. Two buds were grafted on the individual rootstock shoot to ensure the production of a composite plant (Figure 1C). Grafted plants were further cultivated in a greenhouse as described (Sidorova et al., 2019), subjected to chilling during the winter, and allowed to develop shoots from the grafted scion buds (Figure 1D). Prior to inoculation, one of the scion shoots (weaker) was cut off according to the common grafting practice.

### Analysis of Infected Plants

At the first stage of research, before creating transgrafted plum trees, it was necessary to make sure PPV-infected transgenic lines RNAi1 and RNAi2 of rootstock “Elita” were tested for virus accumulation through observation of symptoms, standard Double Antibody Sandwich ELISA (DAS-ELISA), and reverse transcription PCR (RT-PCR). Leaf samples were collected from infected plants and from shoots developed from grafted buds (if available) three times per growing season. ELISA was carried out as described (Sidorova et al., 2019) using a Loewe Biochemica kit (Loewe Biochemica GmbH, Germany). Absorbance values at 415 nm were measured using an iMark Microplate reader (Bio-Rad, United States).

For quantitative RT-PCR (qRT-PCR) analysis, total RNA was isolated from leaves as described (Meisel et al., 2005). cDNA was generated from 5 μg of the total RNA using RevertAid Reverse Transcriptase (Thermo Scientific, Lithuania). Viral genomic components including helper component proteinase (*HC*-*Pro*) and RNA-dependent RNA polymerase (*RdRp*) genes were detected using two sets of primers. For detection of a 442 bp fragment of *HC-Pro* gene, the primers 5'-gtc-tct-tgc-aca-aga-act-ata-acc-3' and gta-gtg-gtc-tcg-gta-tct-atc-ata-3' were used. Amplification of a 1018 bp fragment of *RdRp* gene was performed using specific primers 5'-gaa-gga-aat-ttg-aaa-gca-gtt-ggagc-3' and 5'-cat-tca-cra-art-acc-grc-aaa-tgc-a-3'. To verify the RNA quality and reaction efficiency, primers specific to the endogenously expressed plum *actin1* gene, 5'-tgt-ggc-tcc-aga-aga-aca-tcc-agt-tc-3' and 5'-gaa-aag-tac-ttc-tgg-gca-gcg-gaa-ac-3', were used.

Western blot analysis was carried out using rabbit polyclonal antibodies (IgG) to PPV coat protein from the Loewe Biochemica kit (Loewe Biochemica GmbH, Germany); antibody was diluted 1:500. Anti-rabbit IgG conjugates with alkaline phosphatase (1:3000; Pierce, United States) was used as secondary antibody. The total protein solution (35 μl) from the leaves of analyzed plants was extracted as described (Sidorova et al., 2019), separated on 12.5% SDS-PAGE, and transferred onto NC membrane (Bio-Rad, United States) by tank transfer. The membrane images were developed by a Novex®AP chromogenic substrate (Thermo Fisher Scientific, United States).

*Plum pox virus* accumulation in tissues of composite and transgrafted plum plants was evaluated by ELISA and qRT-PCR. Leaf samples for ELISA were collected three times after 1.5 months of cold-induced dormancy in February, March, and April 2020 from all inoculated plants. Depending on the availability of leaf material, leaf extracts were prepared from the scion part, the rootstock part, and shoots (noted as “graft”) developed from grafted infected buds.

Viral RNA accumulation was studied with qRT-PCR in triplicate using SYBR Green qPCR SuperMix (Thermo Fisher Scientific) and run on a QuantStudio™ 5 Real-Time PCR System (Applied Biosystems). Specific primers for viral RNA detection were 5'-tcg-gac-cca-atg-caa-gtg-ta-3 and 5-gtt-tgc-ctg-ggt-cgg-agt-ag-3'. The results were normalized to the housekeeping gene *Nad5* using the primers 5'-gat-gct-tct-tgg-ggc-ttc-ttg-tt-3' and 5'-ctc-cag-tca-cca-aca-ttg-gca-taa-3' (Menzel et al., 2002). Raw PCR data were analyzed using QuantStudio TM Design and Analysis (Applied Biosystems, Thermo Fisher Scientific, United States).

### Small RNA Analysis

Total RNA was extracted from young leaves (1 g) using an acid guanidinium thiocyanate-phenol-chloroform extraction method as described (Chomczynski and Sacchi, 2006). To enrich the fraction of sRNA, extracted total RNA was incubated overnight at 4°C with 4М LiCl (1:1 v:v) solution and then centrifuged (10,000 *g*) for 15 min at 4°C to precipitate the high-molecular-weight nucleic acids. The remaining supernatant containing the low-molecular-weight RNA was then mixed with cold-ice 96% (v/v) ethanol and stored overnight at −20°C. The low molecular weight RNA was collected by centrifugation at 10,000 *g* for 15 min at 4°C and washed twice with 80% (v/v) ethanol, and the remaining pellet was then resuspended in diethyl pyrocarbonate (DEPC)-treated water.

Libraries of sRNA were prepared using the Illumina TruSeq Small RNA Sample Preparation Kit (Illumina, United States). The pooled libraries were sequenced (read length 50 bp) using the Illumina HiSeq 2500 platform. The number of obtained sRNA reads for each sample is presented in Supplementary Table 1.

The quality of sRNA reads was checked by FastQC^1^ and high-quality reads were aligned to the PPV RNA genome sequence (NCBI accession number: D13751.1) and to the hairpin PPV-RNAi sequence. Alignment was carried out by ShortStack 3.8.5 (Axtell, 2013) with default settings. For analysis of *de novo* annotated small RNA clusters, data from the “Counts” ShortStack file were used. To determine the distribution of sRNA along the PPV-hpRNAi construct and PPV RNA genome sequences, sRNA reads of individual samples were aligned by ShortStack 3.8.5 (Axtell, 2013) to get BAM files. Then, the BAM files were used for coverage calculation using the bedtools coverage command (Quinlan, 2014) with “-d –s” parameters. Data visualization was performed in Rstudio version 1.2.1335^2^ with R version 3.6.0. using ggplot2^3^ and ComplexHeatmap (Gu et al., 2016) packages.

To identify the endogenous small RNA transferred from transgenic “Elita” rootstocks to non-transgenic “Startovaya” scions, several criteria were used: (1) The expression of transferred sRNA in samples of wild-type rootstock “Elita” and two transgenic “Elita” lines was above the median expression of all sRNAs in the corresponding samples. (2) The expression of transferred sRNA was discovered in all samples, including wild-type “Elita,” two transgenic “Elita” lines, and four “Startovaya” scions grafted on transgenic “Elita” lines. (3) sRNA expression was not detected in the wild-type “Startovaya” sample. (4) The expression of transferred sRNA in four “Startovaya” scions grafted on transgenic “Elita” rootstocks lines was above the median expression of all sRNAs detected in wild-type “Startovaya.” Rootstock “Elita”-specific sRNA clusters were defined as those with above the median expression in all rootstock samples, including wild-type “Elita” and two transgenic “Elita” lines, RNAi1 and RNAi2. Detailed information concerning the analyzed sRNA samples is presented in Supplementary Table 1.

## Results

### Evaluation of Resistance to PPV in Transgenic Plum Rootstock Lines

**Figure 2:**
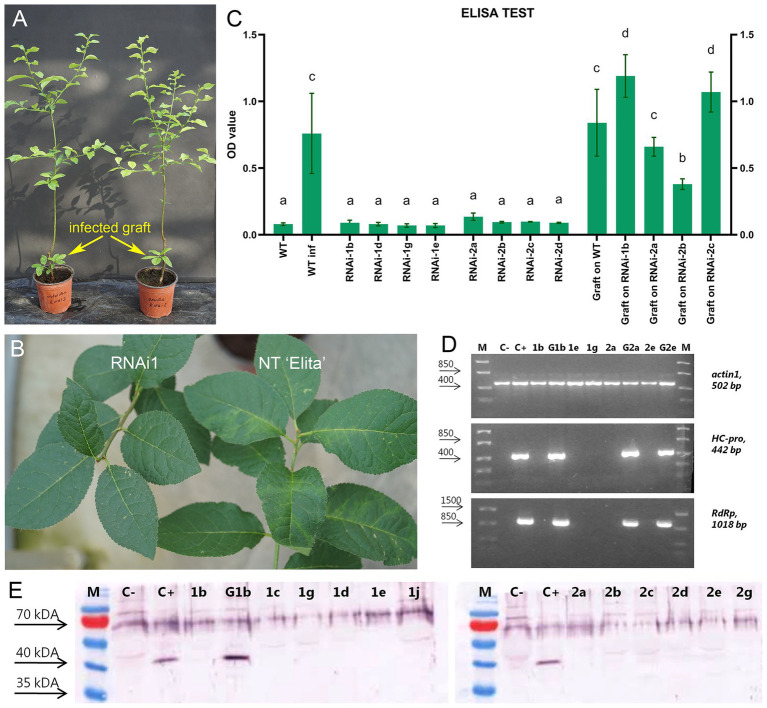
Analysis of transgenic lines of plum rootstock “Elita” for resistance to PPV. **(A)** Two transgenic plum trees (RNAi2) with infectious grafts developed from infected buds, during second vegetative season after inoculation with PPV by T-budding. **(B)** Visual monitoring for symptoms of PPV infection on transgenic (RNAi1) and non-transgenic (NT “Elita”) rootstock plants 2 years after viral inoculation. **(C)** Double Antibody Sandwich ELISA (DAS-ELISA) assay applied to leaves of PPV-inoculated plants. WT, uninfected wild-type rootstock; WTinf, PPV-infected non-transgenic rootstock plant. 1b, 1c, 1g, 1e, and 1j are individual plants of transgenic line #RNAi1; 2a, 2b, 2c, and 2d are individual plants of line RNAi2. OD values are presented for infectious shoots (“infected grafts”) grown from infected buds on WT and various individual transgenic plants of RNAi1 and RNAi2 lines. Means with the same letter in the column have no significant differences according to Duncan’s multiple range test (*p* < 0.05); **(D)** Example of reverse transcription PCR (RT-PCR) assay of transgenic plum rootstock lines infected by PPV. Amplification of specific fragments of PPV *HC-Pro* (442 bp) and *RdRp* (1018 bp) genes is shown; endogenous plum *actin* gene (502 bp) is used as reference gene. M, DNA marker; C−, uninfected rootstock plant; C+, infected wild-type plant. 1b, 1e, and 1g are individual plants of transgenic rootstock line RNAi1; 2a and 2e are individual plants of transgenic rootstock line RNAi2; G1b is infectious graft on RNAi1 1b plant; G2a and G2e are infectious grafts on RNAi2 2a and 2e plants. **(E)** Western blot analysis of protein extracts collected from transgenic plum rootstock plants using an antibody to PPV *CP*. M, marker; C−, uninfected rootstock plant; C+, infected wild-type plant. 1b, 1c, 1g, 1e, and 1j are individual plants of transgenic rootstock line RNAi1 infected with PPV; 2a, 2b, 2c, 2d, 2e, and 2e are individual plants of transgenic rootstock line RNAi2 infected with PPV; G1b is infectious graft on RNAi1 1b plant.

At the first stage of research, before creating transgrafted plum trees, it was necessary to make sure that the previously generated transgenic rootstock “Elita” was reliably resistant to PPV. To protect rootstock plants from viral attack, the pCamPPVRNAi vector, containing an intron-spliced sequence of the PPV CP protein (Dolgov et al., 2010), previously used to produce PPV-resistant “Startovaya” plants (Sidorova et al., 2019), was introduced into the “Elita” genome. Two independent transgenic lines, RNAi1 and RNAi2, were generated *via Agrobacterium*-mediated transformation of leaf explants (Sidorova et al., 2018) to retain the original features of the rootstock. To evaluate viral resistance, the greenhouse-grown transgenic RNAi1 and RNAi2 lines were challenged with the PPV-M strain (NCBI accession number: AJ243957.1) by graft inoculation of 1-year-old plants (Figure 2A).

After the first cold-induced dormancy, visible symptoms of virus infection were found on the leaves of surviving infected grafts and the leaves of non-transgenic “Elita” plants (Figure 2B) challenged in parallel with the transgenic rootstock. All individual transgenic plants of RNAi1 (*n* = 9) and RNAi2 (*n* = 8) infected with virus-containing buds did not display the characteristic symptoms of PPV infection (Figure 1B) during the two vegetative seasons. After the second cold-induced dormancy, four of the individual infected RNAi1 and RNAi2 plants were analyzed by ELISA, showing nearly equally low optical density (OD) values for infected transgenic and non-infected NT “Elita” plants. In contrast, the protein extracts taken from leaves of infected non-transgenic “Elita” plants and from leaves of surviving infected grafts showed significantly higher OD values (Figure 2C).

Viral infection was also tested by RT-PCR (Figure 2D) using the primers designed for the detection of fragments of viral genes, such as *HC-Pro* and *RdRp*. The presence of the viral genome in total RNA extract was confirmed only in the sample of infected non-transgenic “Elita” plant and in samples of surviving infected grafts (Figure 2D). Western analysis with an antibody directed against the coat protein of PPV showed that it was not detectable in the protein extracts of all infected plants of RNAi1 and RNAi2 events, but the PPV virion was abundant in the PPV-positive non-transgenic “Elita” and in the graft grown from infected bud (Figure 2E). The healthy phenotype observed in infected rootstock plants expressing hpRNAi construct allowed us to start studying viral resistance in transgrafted plum plants.

### Virus Resistance Assay of Transgrafted Plum Trees

**Figure 3:**
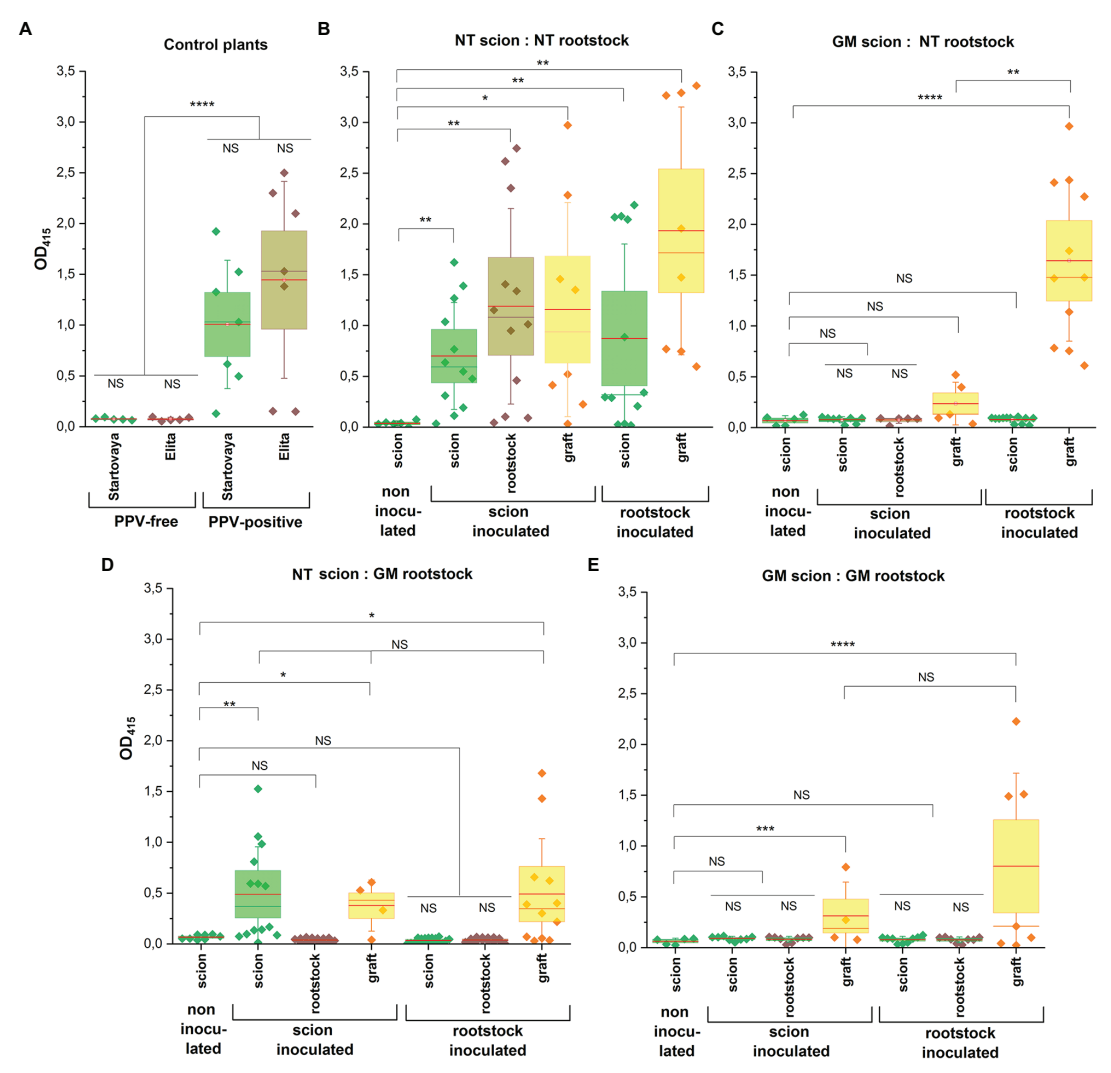
Detection of PPV accumulation in composite and transgrafted plum trees by ELISA. Leaf samples were collected three times after 1.5 months post-cold-induced dormancy in February, March, and April 2020 from all inoculated trees. Depending the availability of leaf material, leaf extracts were prepared from scion part, rootstock part, and shoots developed from grafted infected buds. Four combinations were included: **(B)** non-transgenic (NT scion:NT rootstock) trees; **(C)** transgrafted trees consisting of transgenic “Startovaya” line as scion and non-transgenic “Elita” as rootstock (GM scion:NT rootstock); **(D)** non-transgenic “Startovaya” scion grafted on transgenic “Elita” rootstock (NT scion:GM rootstock); and **(E)** completely transgenic combination of “Startovaya” and “Elita” (GM scion:GM rootstock). **(A)** Additionally, PPV-free and PPV-infected plants of cvs. “Startovaya” (scion) and “Elita” (rootstock) were used for comparison. Plants of each combination were challenged with PPV by T-budding on either scion or rootstock. All transgenic material of “Startovaya” and “Elita” expressed the same hpRNA construct consisting of self-complementary intron-separated fragments of PPV-CP gene sequence driven by enhanced 35S promoter. In each of scion:rootstock combinations, OD values were compared with average OD value of samples taken from non-inoculated scion through an unpaired *t*-test with Welch’s correction: ^*^p < 0.05, ^**^p < 0.01, ^***^p < 0.001, and ^****^p < 0.0005; NS, non-significant.

Two types of transgrafted trees were designed. Attention was mainly focused on to the plum plants when wild-type NT scion (cv. “Startovaya”) was grafted on the PPV-resistant transgenic rootstock (RNAi1 “Elita” event). Additionally, trees consisting of the transgenic scion (RNAi1 “Startovaya” line) grafted on the NT rootstock (cv. “Elita”) were designed. All transgenic parts of transgrafted trees, both scion and rootstock, expressed the same hpRNAi construct. For comparative evaluation, two groups of composite trees were also produced. One of the represented plants consisting of NT scion and NT rootstock, and the other consisted of composite trees where both scion and rootstock were transgenic. Overall, 28 transgrafted and composite trees were produced (Supplementary Table 2); 20 trees were challenged with PPV by inoculation of either the scion or the rootstock (Figure 1B). Ideally, the analyzed trees consisted of the three types of branches representing scion, rootstock, and the graft grown from the PPV-infected bud (Figure 1F). In some trees, however, the original branches of rootstocks did not survive (Figure 1E) after the two rounds of grafting (first by scion and then by infected buds). As expected, the grafted infected buds usually sprouted poorly or produced only a few leaves. Altogether, this restricted the sampling for ELISA tests from rootstocks and grafts during the entire growing season, while scion samples were constantly available from all the trees. Collectively, ELISA tests were performed three times during the vegetation period using all available leaf material. The data summarized across the four scion:rootstock combinations are presented in Figure 3.

As expected, unambiguous signs of PPV infection were found on leaves of the NT:NT (scion:rootstock) combination, where all parts of the tree were non-transgenic. Despite significant seasonal fluctuations of the individual values in some samples, infection was confirmed by high ELISA values in all analyzed extracts including scions, rootstocks, and grafts (Figure 3B). Regardless the type of inoculation (scion or rootstock), the OD values were very close to the values detected in the leaves of previously infected PPV-positive “Startovaya” and “Elita” trees (Figure 3A). It was evident that the virus was easily transmitted into NT rootstock after the grafting of infected buds on NT scion. Even though we could not test the inoculated rootstock because of the lack of branches, ELISA of scion leaves revealed a clear viral spread into the upper part of the tree (Figure 3B). Altogether, the data confirm the bi-directional transmission of PPV in experimental NT:NT plum trees.

Periodic ELISA sampling of completely transgenic trees found that no virus transmission occurred from inoculated scion and inoculated rootstock (Figure 3E). The rootstock and scion remained free of infection and displayed low OD values, equal to uninfected plants. At the same time, an increased ELISA absorbance value was present in the leaves of infected grafts.

Transgrafting scion expressing the hpRNAi construct onto NT rootstock (combination B) prevented virus transmission from the PPV-inoculated scion, since PPV was not detected in the rootstock during the growing season, either visually or by ELISA test (Figure 3C). When NT rootstock was inoculated with PPV, the transgenic scion remained ELISA-negative, while the graft displayed very high OD values. Unfortunately, the NT rootstocks challenged with the virus did not produce branches, so the data concerning the virus accumulation in the leaves of NT rootstock are not available. Once during the growing season, bark samples were taken from NT rootstocks of the trees B4 (inoculated GM scion) and B2 (inoculated NT rootstock) and analyzed serologically for viral infection. ELISA failed to detect PPV in the tissue of the B4 rootstock (OD_415_ = 0.09), while in the stem tissue of the B2 rootstock the accumulation of virus was confirmed by higher ELISA values (OD_415_ = 0.39).

The analysis of transgrafted trees consisting of GM rootstock and NT scion (combination D) showed that the rootstock-to-scion movement of PPV did not occurr. Even under the permanent input of the virus from infected grafts, the inoculated transgenic rootstock showed no ELISA-detectable level of infection (Figure 3D). Consequently, the NT scions also remained ELISA negative and showed no signs of Sharka disease in the case of inoculation of the GM rootstock. When NT scions of NT:GM transgrafted trees were infected with PPV-containing buds, typical disease symptoms appeared on the leaves of NT scions. There was no clear difference in PPV incidence among infected NT scions (Figure 3D) and the PPV-positive “Startovaya” tree used as a source of infected buds (Figure 3A). The mean ELISA values for leaf samples of NT scion were 0.44 (tree D4) and 0.65 (tree D5), fluctuating above 1.00 in individual samples, which was significantly higher the threshold level. The observed ELISA absorbance values were similar to those of the PPV-positive “Startovaya” tree displaying obvious viral symptoms over several years (OD_415_ = 0.99), as well as those of NT scions of NT:NT combination infected in the same way (OD_415_ = 0.69; Figure 3B). Nevertheless, the transgenic rootstock of transgrafted NT:GM trees were ELISA negative (OD_415_ = 0.04) throughout the time of sampling.

**Figure 4:**
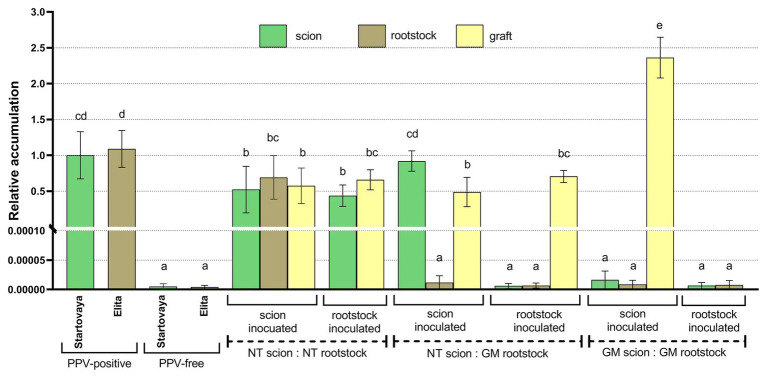
Relative quantification of accumulation of PPV RNA in total RNA samples extracted from composite and transgrafted plum trees representing three scion:rootstock combinations. Leaf samples were randomly collected in the middle of vegetative period from trees A6, A7, D4, D2, C5, and C1. PPV-positive “Startovaya” extracts served as reference samples. Statistical significance of differences (*p* < 0.05) between groups was tested using one-way ANOVA. Means with the same letter in the column have no significant differences according to Turkey’s multiple range test (*p* < 0.05).

To validate the data of ELISA analysis, leaf samples from three scion:rootstock combinations (NT:NT, NT:GM, and GM:GM) were randomly chosen from trees A6, A7, D4, D2, C5, and C1 for extraction of total RNA to conduct RT-qPCR analysis, a technique that is much more sensitive in detecting PPV than ELISA. As the viral RNA is not normally present in the plant cells, the PPV-positive “Startovaya” extracts served as reference samples. As expected, RT-qPCR confirmed the presence of viral RNA in all analyzed samples taken from NT:NT trees; the level of viral RNA accumulation in the scion, rootstock, and graft did not differ statistically from that of PPV-positive “Startovaya” and “Elita” trees (Figure 4). By RT-qPCR, viral RNA was not present in all transgenic parts of NT:GM and GM:GM trees. The relative level of PPV RNA accumulation was thousands of times lower than in the PPV-positive reference samples, and was not significantly different from that of PPV-free “Startovaya” and “Elita” trees (*p* > 0.01). The analysis of transgrafted trees showed that the transgenic rootstock, expressing the self-complementary hpRNA construct was a reliable barrier for the systemic spread of PPV, since the accumulation of viral RNA was found only in shoots grown from PPV-infected buds grafted on the GM rootstock. At the same time, overexpression of the PPV-hpRNAi construct by cells of the transgenic rootstock did not promote the PPV resistance in the NT scion. According to RT-qPCR data, the level of viral RNA accumulation in the leaves of NT scion inoculated with PPV was as high as in the samples of the PPV-positive “Startovaya” tree (Figure 4).

### Analysis of Transgene-Induced and Endogenous sRNA

**Figure 5:**
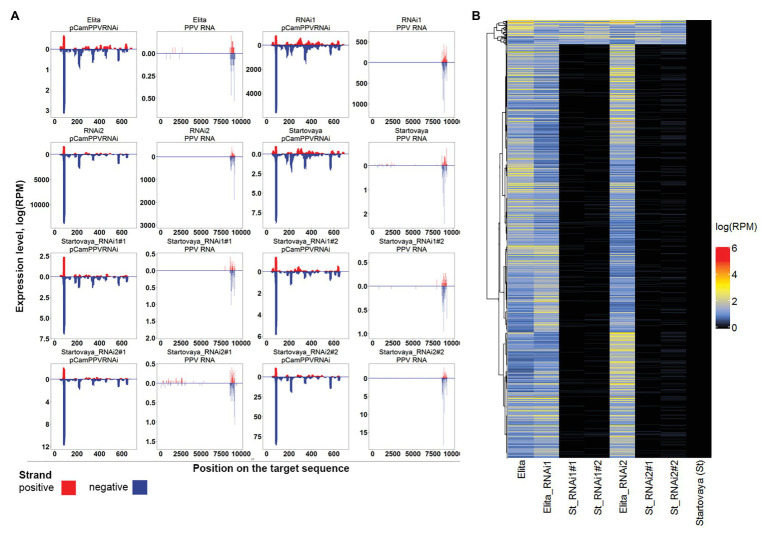
Transgenic-derived and endogenous small RNA (sRNA) profiling. **(A)** Distribution of sRNAs along PPV-hpRNAi construct and PPV-D RNA genome (PPV RNA) sequences. sRNAs of eight samples are shown including two non-transgenic cultivars, “Startovaya” and “Elita,” two transgenic rootstock “Elita” lines (Elita_RNAi1 and Elita_RNAi2), and four “Startovaya” non-transgenic scions grafted on transgenic rootstocks Elita_RNAi1 (St_RNAi1#1 and St_RNAi1#2) or Elita_RNAi2 (St_RNAi2#1 and St_RNAi2#2; NT scion: GM rootstock transgraft combination). **(B)** Heatmap of expression values of rootstock-specific endogenous sRNAs in samples described in **(A)**.

As the presence of specific sRNA is the hallmark of the activation process of viral RNA silencing, we further analyzed the profiles of transgene-induced siRNA by sequencing sRNA pools derived from non-infected NT scions grafted on transgenic rootstocks. Samples of sRNA pools were taken from NT scions (“Startovaya”) grafted on two transgenic “Elita” rootstocks, RNAi1 and RNAi2. Samples were collected from two independently transgrafted plants of each transgenic event. The sRNA reads were mapped to the PPV-genomic RNA and the hairpin PPV-RNAi sequence of pCamPPVRNAi vector. The profiles were compared with sRNA profiles of transgenic RNAi1 and RNAi2 events, and with those of non-transformed “Elita” and “Startovaya” plum trees (Figure 5A).

The analysis revealed that both GM rootstock lines successfully generated transgene-derived sRNA due to overexpression of the introduced hairpin cassette. In total, 20380.0 and 13273.5 reads per million sequencing reads (rpm) of sRNA (20–24 nt) specific to the hairpin construct and genomic RNA of PPV, respectively, were detected in the sRNA pool of the RNAi1 event (Table 1). The most prevalent sRNA type was 21 nt, constituting 73% of the total mapped sRNA reads. For RNAi2, higher amounts of specific sRNA were detected, 30547.6 rpm for the PPV-hpRNAi construct and 15509.6 rpm for the PPV RNA genome. In both transgenic lines of rootstock, the distribution of sRNA along the target sequences was random relative to the introduced hairpin construct, with a prevalence of reverse sequences (Figure 5A). The mapping showed that position 70–95 nt on the hairpin arm yielded the most abundant sRNA (Figure 5A). In general, the same peak was characteristic for all samples, but the total number of reads in extracts of RNAi1 and RNAi2 “Elita” events was several orders of magnitude higher than in extracts of non-transformed “Elita” (Figure 5A). Concerning the genomic RNA of PPV, reads were distributed in the 8579–9272 bp region specific to the CP gene sequence, which was used as an arm for designing the intron-spliced hairpin structure in the pCamPPVRNAi vector.

**Table 1:** Number of sRNA reads from eight samples^*^ of sRNA pools aligned to hpRNAi construct and PPV-D RNA genome sequences.

Target sequence	“Elita”	“Startovaya”	Elita_RNAi1	Elita_RNAi2	St_RNAi1#1	St_RNAi1#2	St_RNAi2#1	St_RNAi2#2
PPV-D RNA	5.3	18.2	13273.5	15509.6	11.2	10.1	13.4	112.5
hpRNAi	10.6	29.3	20,380	30547.6	21.2	16.9	26.6	210.8

* Eight samples of sRNA pools were extracted from two non-transgenic plants of cultivars “Startovaya” and “Elita,” two transgenic “Elita” lines (Elita_RNAi1 and Elita_RNAi2), and four samples of non-transgenic “Startovaya” scions grafted on transgenic rootstocks Elita_RNAi1 (two samples St_RNAi1#1 and St_RNAi1#2) or Elita_RNAi2 (two samples St_RNAi2#1 and St_RNAi2#2).

The sRNA pools derived from extracts of non-transformed “Elita” and “Startovaya” plants contained 10.6 and 29.3 rpm, respectively, of endogenous sRNA coinciding with regions of the hpRNAi construct. A few reads were also mapped to the regions of PPV RNA sequence, including 5.3 and 18.2 rpm in sRNA pools of “Elita” in sRNA pool of “Startovaya,” respectively (Table 1). The low portion of such reads in non-transgenic plum samples indicates that the sequencing errors are reliable for profiling transgene-derived siRNA.

The sequencing of sRNA pools extracted from two NT scions grafted on the RNAi1 rootstock plants showed the presence of 10.1–11.2 rpm matching the PPV RNA genome and 16.9–21.2 rpm matching the intron-spliced hairpin structure (Table 1). It was evident that the number of sRNA reads mapped to hairpin arms of a vector remained low, as the same amount (or even more) was found in the extracts of non-transgenic “Elita” and “Startovaya” plants. Analysis of sRNA pools isolated from the two non-transformed scions (samples 1 and 2) grafted on the RNAi2 event revealed some discrepancies between the two analyzed samples. While sample 1 showed a low siRNA level (26.6 rpm), the accumulation of siRNA in sample 2 was nearly 8 times higher (210.8 rpm; Figure 5A; Table 1). In any case, the hpRNA-specific siRNA represented only 0.005% of the overall sRNA pool of the NT scion, while the portion of transgene-derived sRNA in transgenic rootstock line RNAi2 was more than 2%. Thus, the transfer of transgene-derived sRNA from rootstock to scion was negligible in our experiments.

Since the scion (“Startovaya”) and the rootstock (“Elita”) of transgrafted trees belonged to different plum species, we examined the possibility of transferring the rootstock-specific endogenous sRNA into the scion using the data from *de novo* annotation of small RNA clusters. A total of 78,651 sRNA clusters were identified, and 1,287 of them were expressed with >1 rpm exclusively in rootstock samples, both GM and NT (Figure 5B). Comparable analysis of sRNA pools of scions and rootstocks revealed that sRNAs from 78 (5.1%) “Elita”-specific clusters were detected in scions, suggesting that these sRNAs were transferred from the rootstock (Figure 5B). Hence, the rootstock-to-scion sRNA transfer was not blocked in the analyzed transgrafted plants.

## Discussion

In plants, various multiple silencing factors may participate in antiviral defense (Duan et al., 2012). Among them, the mobile siRNA signal has been proved to play a critical role in protecting plant cells from attacks of viral RNA molecules (Khalid et al., 2017). With the help of dsRNA-producing construct targeting the specific PPV sequence, we significantly elevated the amount of virus-specific siRNA in transgenic rootstock “Elita.” Accumulating in the cells to a level of 2% of the total amount of sRNA, the transgene-derived siRNA conferred durable resistance to PPV. Previously, the same construct also provided unbreakable viral resistance for European plum “Startovaya” (Sidorova et al., 2019), indicating that the siRNA signals produced by this construct can be universally used by various transgenic plum species and varieties. The encouraging results prompted us to create plum trees combining transgenic/non-transgenic tissues of “Startovaya” and “Elita.” Despite a certain limitation in the number of individual trees infected with the virus, the expression of silencing siRNA transcripts in transgenic tissues was undoubtedly a reliable barrier to the further spread of the virus to NT graft partners. The accumulation of virus-specific siRNA in transgenic scion or transgenic rootstock effectively prevented the movement of virus in both directions, from the inoculated GM scion to the NT stock, and from the inoculated GM stock to the NT scion. The observed results cannot be related to the combinatorial effects between scion and rootstock, since the virus did not encounter any obstacles to spreading when analogous non-transgenic trees were infected in either the upper or lower parts.

The dsRNA/siRNA silencing signals produced by transgenic tissues have repeatedly been reported to show mobility through graft unions in herbaceous and woody plants (Pyott and Molnar, 2015; Kyriacou et al., 2017; Kehr and Kragler, 2018; Liu and Chen, 2018; Choudhary et al., 2019; Zhang et al., 2019). Based on this, we expected to trigger RNAi-mediated resistance in inoculated NT scion through grafting on transgenic rootstock, abundantly accumulating transgene-derived siRNA molecules. In the present study, repeated molecular and visual diagnosis could not verify that the leaves of NT scions from transgrafted plum trees displayed any signs of resistance, even partial. Virus particles easily migrated from infected grafted buds to the NT scion. The level of viral accumulation, detected by ELISA and RT-qPCR, was in general equal to the viral infection of NT trees and separately infected control plants. Similarly, when NT rootstock was combined with GM scion, the reverse transgrafting also failed to protect inoculated NT rootstock from the viral infection.

Small RNA deep sequencing indicated that most of the analyzed NT scions of transgrafted trees had a very low level of transgene-induced siRNA. The lack of PPV resistance was due to the insufficient transfer of transgene-derived silencing signal from GM rootstock to NT scion. The minor translocation of transgene-specific siRNA was detected in one of the NT scion samples, but it also seemed to be insufficient to stimulate active RNAi-mediated silencing and protect NT tissues from viral attack in plum. This result contradicts the research of Zhao and Song (2014), who reported that transgene-derived mobile siRNA silencing signals of a GM rootstock were able to protect NT scion of sweet cherry from infection with PNRSV. In sweet cherry, close relative woody species, the graft-transmissible movement of PNRSV-hpRNA-derived siRNAs (20–21 nt) reached 187–199 reads. The accumulation of construct-specific siRNA in transgenic plum rootstock was significantly higher (20,380–30,547 rpm) than in transgenic cherry rootstock (1,925–2,856 rpm); however, transitive silencing in plum was not achieved. At the same time, the transgenic rootstock demonstrated the ability to transport into the scion tissues a pool of various unique sRNAs, which are characteristic to the rootstock “Elita” and not naturally produced by “Startovaya.” At the very least, this observation indicates that the transport of endogenous sRNA from the rootstock to the scion occurred in our study at a high level, which is equal to that observed in recent research examining heterografts between highly compatible scions and rootstocks of sweet cherry (Zhao et al., 2020).

Our results are more consistent with those of Flachowsky et al. (2012), who reported that the graft transmission of RNAi silencing signal did not occur in greenhouse-grown apple (*Malus* spp.), another member of the *Rosaceae* woody tree family. Similarly, in walnut, mobilization of transgenic siRNA across the graft into NT leaves was also not detected (Haroldsena et al., 2012). Flachowsky et al. (2012) hypothesized that the physiological and morphological transition from herbaceous-like tissues (*in vitro*) to a woody-like tissues (*ex vitro*, greenhouse) due to lignification process would negatively influence the systemic transport of silencing signals. This factor can certainly influence the transitivity, as various transgrafted woody species, including plum, had apparent problems with the transition of siRNA and other transgene-derived content (protein and mRNA) across the graft union (Nagel et al., 2010; Smolka et al., 2010; Haroldsena et al., 2012; Liu et al., 2017). A lack of siRNA transitivity was also reported for herbaceous transgfrafts of tomato and tobacco (Haroldsena et al., 2012; Tzean et al., 2020). No doubt, the contradictory data can be attributed to the differences between plant species, the transgenic construct, and the targeted sequence (exogenous infecting virus or endogenous gene transcripts). However, those common factors do not explain the lack of long-distance communication between graft partners in plum concerning transgene-derived siRNA populations.

Evidence from previous transgrafting reports indicates that maintaining some leaves in a transgenic rootstock is necessary to provide mobility of transgene-derived molecules such as *FLOWERING LOCUS*-induced florigenic signals. Unsuccessful attempts to alter the scion flowering regime in various species were associated with transgrafting on leafless GM rootstocks (Wenzel et al., 2013; Walworth et al., 2014; Bull et al., 2017). When the leafy or branched plants of transgenic tobacco (Walworth et al., 2014), jatropha (Ye et al., 2014), and blueberry (Song et al., 2019) were used as graft rootstocks, the systemic signal that regulates growth and flowering time was successfully achieved in NT scions. The cultivation of traditional woody trees suggests that only scions develop branches, while rootstocks never produce leaves. In our study, a high accumulation of transgene-derived siRNA was confirmed in the extract of the branched transgenic rootstock. The rootstock part of transgrafted plum plants did not produce a lot of leaves, since only one branch was left for the experiments, if available. Although we used the strong constitutive CaMV 35S promoter to express hairpin construct in all plum tissues, it can be assumed that the expression level in nearly leafless rootstock was not sufficient to provide enough mobile silencing signals. Unfortunately, we could not confirm or disprove this supposition, as the reverse transgrafted trees (GM scion:NT rootstock) lacked rootstock branches to analyze scion-to-rootstock transport.

At the same time, our long experience with transgenic PPV-resistant trees of “Startovaya” showed that the PPV-infected NT branches grafted on the GM plants had never recovered from Sharka disease. The presence of a significant number of transgenic leaves did not prevent the annual appearance of viral symptoms on infected NT grafts even after a decade of solid connection between the vascular systems of mature GM trees and infected NT grafts (Sidorova et al., 2019). It is unlikely that more leaves producing PPV-specific dsRNA precursors can completely cure heavily infected tissue, but it is likely to reduce the total amount of virus in infected tissues to some extent. This assumption requires further study using transgrafted plants produced in the present study.

Although the mechanisms determining the upward and downward mobile silencing are still largely unknown and may be different in various plant species, numerous independent studies confirmed that transgene-induced silencing signals traveled systemically through the phloem (Pyott and Molnar, 2015; Song et al., 2015; Yang et al., 2015; Kehr and Kragler, 2018). Phloem-mediated signaling is thought to be triggered by the accumulation of dsRNA precursors, that give rise to primary siRNA (Sarkies and Miska, 2014; Pyott and Molnar, 2015; Kehr and Kragler, 2018). In transgenic plum tissues constitutively expressing the dsRNA-eliciting construct, there was no need to translocate siRNA signal over long distances and the primary siRNA was directly involved in resistance to PPV. The primary RNAi signal has also been shown to supply both vasculature-to-epidermis and long-distance silencing movement (Devers et al., 2020). Primary siRNA initiates the production of secondary siRNA, also known as “transitive” siRNA, which acts as a mobile silencing signal transmitted over long distances through the plant vasculature regardless of the sequence of dsRNA precursor, the origin (transgenic, endogenous, or exogenous natural infection), and the prevalent size (21, 22, or 24 nt; Schwab and Voinnet, 2010; Pyott and Molnar, 2015; Kehr and Kragler, 2018; Choudhary et al., 2019). In contrast to primary silencing, transitive silencing requires more coordinated activity of a set of plant genes. The availability of various genetic elements and mediators, such as ARGONAUTE (*AGO*), Dicer-like (*DCL*), RNA-dependent RNA polymerase (*RdRp*), and phloem-resident RNA-binding family proteins, may act as a limiting factor determining the biogenesis and systemic silencing efficiency of primary and secondary sRNAs (Sarkies and Miska, 2014; Kehr and Kragler, 2018; Devers et al., 2020). In the case of viral infection, this process is additionally influenced by various strong suppressors of silencing produced by the penetrated virus (Duan et al., 2012; Devers et al., 2020).

Based on the current knowledge, various approaches could potentially be used in plum to increase the transitivity of silencing signals produced by a transgenic tissue. A recent study on transgrafted tobacco showed that replacing the constitutive viral 35S promoter with the phloem-specific promoter ensures the transitive viral resistance of NT scion (Tzean et al., 2020). Although the phloem-specific phRNA expression construct generated less viral-derived sRNA than the 35S-based one, non-infected plants were observed only when wild-type scions were grafted on transgenic rootstocks producing hpRNA under phloem-specific promoter (Tzean et al., 2020). While this report provides information concerning the herbaceous species model, it suggests that companion phloem-specific promoter could potentially be used in plum as a first step to increase the transitivity of RNAi signaling.

The data from the present study indicate that the experimental approach for combining NT scion with transgenic rootstock producing a bulk of PPV-specific siRNA is not sufficient to achieve the effective mobility of antiviral silencing signal. As a result, the non-transgenic recipient of transgrafted plum tree remains susceptible to viral infection. Based on this, more attention should be paid in future experiments to revealing the regulatory network that controls the long-distance signaling *via* the vascular system to promote antiviral silencing machinery in transgrafted plum.

## Data Availability Statement

The original contributions presented in the study are publicly available. This data can be found at: https://www.ncbi.nlm.nih.gov/bioproject/PRJNA673051.

## Author Contributions

TS and SD conceived the study and produced transgrafted trees. SD supervised the research. TS and AP carried out the molecular analysis. IK carried out the bioinformatics. TS, DM, and IK analyzed the data. DM wrote the paper with assistance of TS and IK. AP and SD contributed to writing and revision. All authors contributed to the article and approved the submitted version.

### Conflict of Interest

The authors declare that the research was conducted in the absence of any commercial or financial relationships that could be construed as a potential conflict of interest.

## Footnotes

^1^http://www.bioinformatics.babraham.ac.uk/projects/fastqc/

^2^http://www.rstudio.com/

^3^https://cran.r-project.org/package=ggplot2
