# Supplementary material for: Effect of Grafting on Viral Resistance of Non-transgenic Plum Scion Combined With Transgenic PPV-Resistant Rootstock
Source: Front Plant Sci. 2021 Feb 1;12:621954. doi: 10.3389/fpls.2021.621954 (PMC7882617; doi:10.3389/fpls.2021.621954)
Supplement: Supplementary file 1 [file Table_1.docx]

Supplementary Material

**Effect of grafting on viral resistance of non-transgenic plum scion combined with transgenic PPV-resistant rootstock**

Tatiana Sidorova*, Dmitry Miroshnichenko, Ilya Kirov, Alexander Pushin, Sergey Dolgov

* Correspondence: sidorovat@rambler.ru

**Supplementary Table 1**. Summary of samples and number of small RNA sequencing reads

| **Sample name** | **Number of reads** | **Type of sample** |
| --- | --- | --- |
| Elita | 14,922,643 | Non-transgenic wild-type cv. ‘Elita’ |
| Startovaya (St) | 14,802,246 | Non-transgenic wild-type cv. ‘Startovaya’ |
| Elita_RNAi1 | 14,556,849 | Transgenic event RNAi1 of ‘Elita’ used as rootstock for non-transgenic ‘Startovaya’ scions (St_RNAi1#1 and St_RNAi1#2 samples) |
| St_RNAi1#1 | 14,642,046 | Non-transgenic ‘Startovaya’ scion grafted on transgenic Elita_RNAi1 rootstock, biological replicate (tree) #1 |
| St_RNAi1#2 | 14,633,801 | Non-transgenic ‘Startovaya’ scion grafted on transgenic Elita_RNAi1 rootstock, biological replicate (tree) #2 |
| Elita_RNAi2 | 15,009,598 | Transgenic event RNAi2 of ‘Elita’ used as rootstock for non-transgenic ‘Startovaya’ scions (St_RNAi2#1 and St_RNAi2#2 samples) |
| St_RNAi2#1 | 14,901,226 | Non-transgenic ‘Startovaya’ scion grafted on transgenic Elita_RNAi2 rootstock, biological replicate (tree) #1 |
| St_RNAi2#2 | 11,392,612 | Non-transgenic ‘Startovaya’ scion grafted on transgenic Elita_RNAi2 rootstock, biological replicate (tree) #2 |

**Supplementary Table 1.** Identification of composite and transgrafted plum trees

| Type of inoculation with PPV | Scion:rootstock combination* | | | |
| --- | --- | --- | --- | --- |
|  | A (NT:NT) | B (GM:NT) | C (GM:GM) | D (NT:GM) |
| 0 (no inoculation) | A1 A3 | B5 B6 | C6 C7 | D1 D3 |
| I (scion inoculation) | A5 A6 | B3 B4 | C1 C2 | D4 D5 |
| II (rootstock inoculation) | A2 A4 A7 | B1 B2 B7 | C3 C4 C5 | D2 D6 D7 |

GM, genetically modified; NT, non-transgenic
